# An integrated petrophysical and rock physics characterization of the Mangahewa Formation in the Pohokura field, Taranaki Basin

**DOI:** 10.1038/s41598-025-88639-4

**Published:** 2025-02-10

**Authors:** Shakhawat Hossain, Naymur Rahman

**Affiliations:** 1 Department of Geology, University of Dhaka, Dhaka, 1000 Bangladesh; 2 Department of Earth Science and Engineering, Imperial College London, London, SW7 2AZ UK

**Keywords:** Energy science and technology, Fossil fuels

## Abstract

The Mangahewa Formation in the Pohokura gas field, Taranaki Basin, is a key reservoir for gas production in New Zealand, yet its deep and heterogeneous nature presents challenges for accurate reservoir characterization. While prior studies have explored aspects of the Mangahewa Formation such as lithology, fluid composition, and petrophysical properties, the interrelationships between these factors and their impact on hydrocarbon production remain underexamined. This study integrates detailed petrophysical and rock physics analyses to overcome these challenges. Petrophysical evaluation, based on well log data from depths of 3200–4000 m, reveals net reservoir thicknesses ranging from 164 to 479 m, with total porosity between 17 and 21% and effective porosity between 8 and 19%. Shale volume and water saturation vary from 21–28 and 22–34%, respectively. Rock physics analysis was performed using Rock Physics Templates (RPTs) to model the elastic properties of the reservoir. The Mangahewa Sandstone exhibits elastic properties consistent with the stiff sand model, with compressional sonic velocities ranging from 4100 to 5000 m/s. High correlations were achieved between measured and modeled velocities, with 97% for V_P_ and 94% for V_S_. These models enabled the estimation of porosity from seismic-derived acoustic impedance, providing valuable insights in areas with limited well control. Furthermore, the RPTs effectively differentiated between gas sand, water sand, and shale facies, minimizing uncertainties in fluid and lithology prediction. These results provide a comprehensive understanding of the Mangahewa Formation, enhancing hydrocarbon prospect evaluation and supporting further exploration and development in the Pohokura field.

## Introduction

Petrophysical analysis is crucial for reservoir characterization, particularly in distinguishing hydrocarbon-bearing zones from non-hydrocarbon zones^1^. Numerous techniques have been developed for fluid and lithology discrimination^2–10^, with petrophysical data often transformed into essential reservoir properties such as porosity, permeability, shale volume, and fluid saturation^11,12^. Accurate analysis of these properties is fundamental in identifying hydrocarbon zones^13^. However, poor borehole conditions, missing logs, and variations in pressure, temperature, and salinity can affect results, while models often fail to account for variability between wells, even those in close proximity^14^.

To address these challenges, integrated workflows combining petrophysical and rock physics analysis have been developed. These workflows allow the creation of consistent rock physics models across reservoirs, enhancing the prediction of elastic logs, well-to-seismic ties, and overall reservoir characterization^15–17^. Calibrated models can reliably predict variations in lithology and fluid saturation^18–20^ and help identify inconsistencies in well log data, improving the accuracy of reservoir evaluation^21^. Rock physics models, which bridge petrophysical properties with seismic data, provide a robust framework for characterizing reservoirs^4,22–24^. Several rock physics models have been proposed, including inclusion models^25^, contact models^26,27^, and models based on bounding and transformation theories^28–30^. Recent work by Sava et al.^31^ demonstrates that rock physics templates (RPTs) can effectively relate fluid properties to seismic responses, allowing for better fluid and lithology discrimination. RPTs have been used to estimate reservoir properties such as porosity, water saturation, and hydrocarbon saturation, reducing uncertainty in reservoir characterization^20^. The application of RPTs and rock physics models has proven especially valuable in complex reservoirs, such as the Thong-Mag Cau formation of Vietnam^32^.

The Mangahewa Formation in the Pohokura gas field, Taranaki Basin, represents a complex and highly heterogeneous reservoir characterized by marginal marine tight sands^33,34^. Previous studies have identified key petrophysical properties, including porosity, permeability and hydrocarbon saturation^33^. However, the diagenetic processes - such as silica cementation and compaction - have significantly reduced porosity and permeability^35^, making it difficult to fully characterize its reservoir quality. The variability in lithofacies further complicates reservoir assessment, with recent studies identifying eight lithofacies, the highest-quality reservoirs being bioturbated, laminated, and massive sandstones^36^.

An integrated petrophysical and rock physics approach is essential to overcome these challenges. Standard petrophysical models may fail to consistently predict properties across the reservoir due to heterogeneity^14,18^. By combining petrophysical analysis with advanced rock physics modeling, this study seeks to provide better correlations between well log and seismic data, improve the spatial resolution of reservoir properties, and reduce uncertainty in fluid and lithology discrimination^19,37–39^. Similar methodologies have been applied successfully in complex reservoirs worldwide, such as the Sawan gas field in Pakistan^6^ and the deep-buried sandstones of the Nam Con Son Basin in Vietnam^32^.

This study aims to deliver a more precise characterization of the Mangahewa Formation using an integrated petrophysical and rock physics workflow. The resulting insights will contribute to more effective exploration and development strategies for the Pohokura gas field.

## Geology of the study area

New Zealand is one of the southernmost countries globally and is located in the southwest of the Pacific Ocean. The country (New Zealand) fragmented into North Island and South Island by 20 km wide Cook Strait which are located in Australian Plate and Pacific Plate respectively^40,41^. Along the southern edge of the South Island, the Pacific Plate thrusts over the Australian Plate. On the eastern edge of the North Island, the Australian Plate thrusts over the Pacific Plate, while in the South Island, the Australian Plate slides past the Pacific Plate, giving rise to a strike-slip fault identified as the Alpine Fault^40^. All these tectonic activities have resulted in the formation of many basins in and around New Zealand.

The famous hydrocarbon-bearing Taranaki Basin is positioned in the western coast of the North Island. Encompassing an area of about 100,000 sq. km, with a significant portion extending into offshore areas. The formation of the Taranaki Basin in the vicinity of New Zealand commenced in the late Cretaceous period through the separation of Australia and Zealandia as part of the Gondwana breakup^43,44^. The configuration of the Taranaki Basin results from three key phases of deformations: firstly, Late Cretaceous to Paleocene rifting, followed by Eocene to Early Oligocene compression, and finally, Oligocene to Recent activity along the active margin^32,45,46^. The structural makeup of the Taranaki Basin is defined by two primary units: the Eastern Mobile Platform and the Western Stable Platform^47^. The Western Stable Platform extends approximately 150 km, stretching from the Cape Egmont Fault in the west to the continental shelf edge and is composed of Late Cretaceous to Paleocene half-graben formations, overlain by deposits formed after the Eocene to Holocene periods. Furthermore, it was influenced by tectonic activity post the Eocene epoch^42,48,49^. The extension of the Eastern Mobile Platform is about 140 km and divided into three units, namely Southern Graben, Central Graben and Northern Graben. The eastern structural unit also comprises the Eastern Tarata Thrust Zone associated with upliftment, Southern Inversion Zone, and volcanic activities^48,50,51^.

**Fig. 1:**
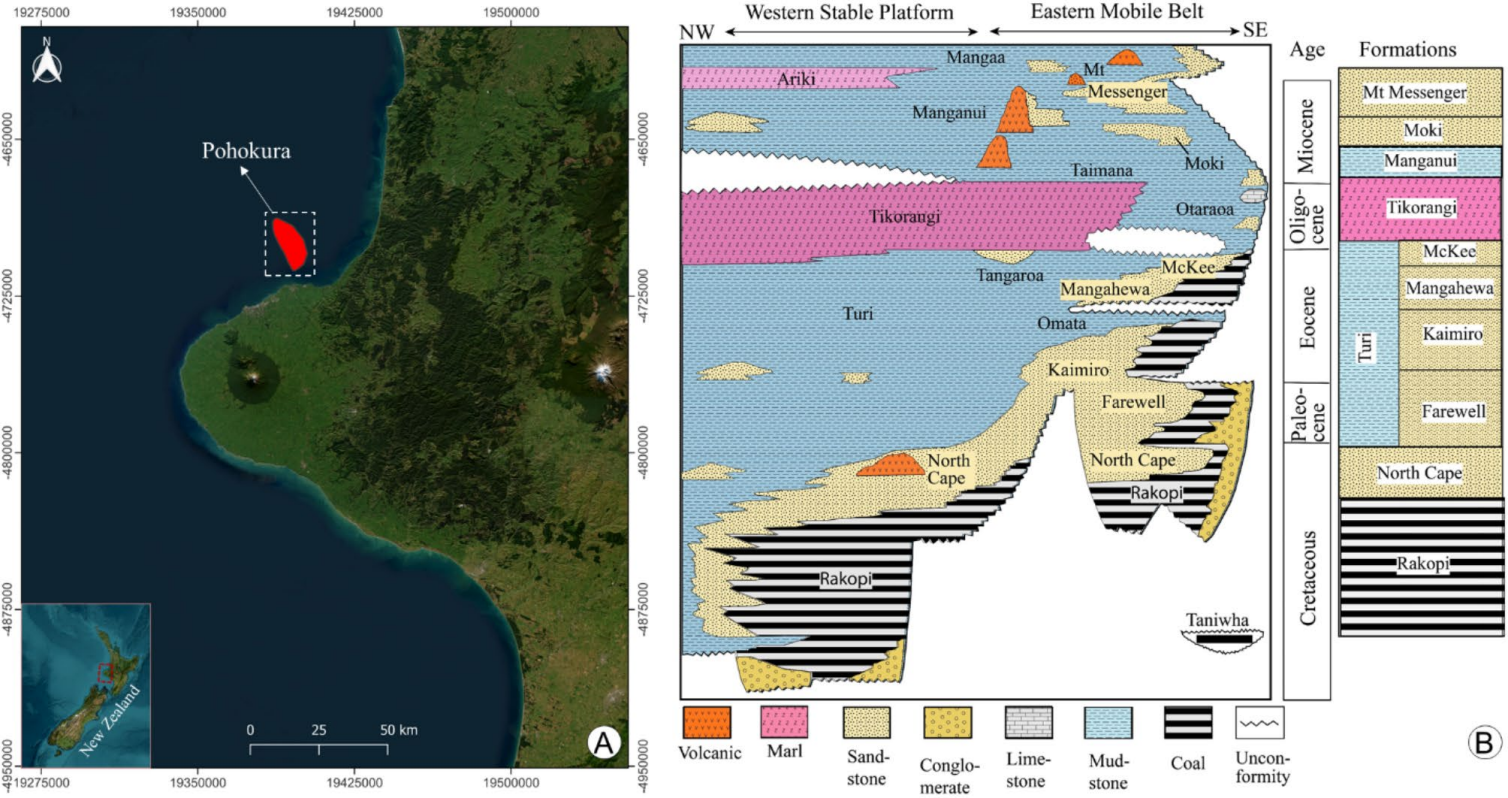
Map displaying the Pohokura Gas Field location (red polygon) (**A**) and the position of the Mangahewa Formation showing in the stratigraphic succession of the Taranaki Basin (**B**) (modified after^42,43^). (**A**) was created using Google Earth imagery (https://www.google.co.uk/earth/) processed in QGIS 3.4 (https://www.qgis.org/download/), while (**B**) was drafted using Adobe Illustrator CC 2021 (https://www.adobe.com/uk/products/illustrator.html).

The Pohokura Field is located within the Northern Graben, positioned at a considerable distance from the northern shoreline approximately 4 km away and lies below 35 m depth of water^52,53^ (Fig. 1A). The Taranaki Fault delineates its eastern boundary, while the western boundary is demarcated by the Cape Egmont Fault^47,54^. Given that the Eastern Mobile Belt contains various compressional and extensional features, which represent the Pohokura structure that has experienced compressional and extensional stresses^51,55^. The geometry of the Pohokura structure is a north-south elongated, low relief anticline with about 16 km in length and 5 km in width in area and about 3600 m in depth^52,53^.

The primary hydrocarbon sources in the Rakopi Formation of the Pakawau Group are the organic marine coal and mudstone^56–58^. Within the Pohokura field, the Mangahewa Formation of Kapuni Group is the thickest (about 280 m thick) and main hydrocarbon bearing zone which comprises coastal sandstone interbedded with siltstone and carbonaceous mudstone and coal^42,45,51,56^ (Fig. 1B). The Turi Formation from the Moa Group acts as the impermeable seal on the Mangahewa reservoir in the Pohokura structure. It comprises micaceous, carbonaceous, dark grey to brown, marine mudstone^42,57^.

## Materials and methodology

### Data conditioning

**Fig. 2:**
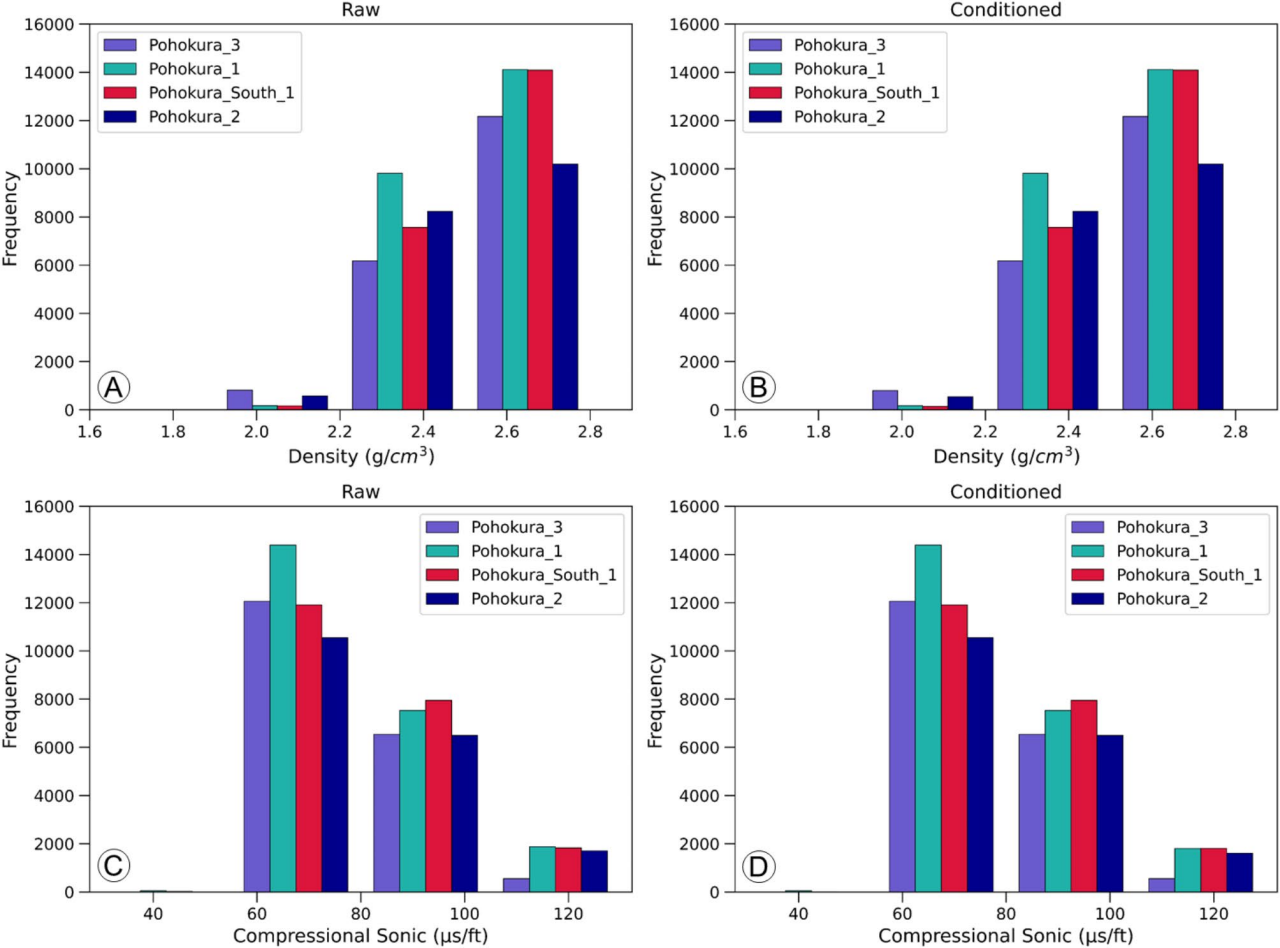
Histograms showing the raw and conditioned density and compressional sonic data. The raw data contains several erroneous intervals (**A**,**C**), while the condition data reflects improved quality after applying the de-spiking method (**B**,**D**).

**Fig. 3:**
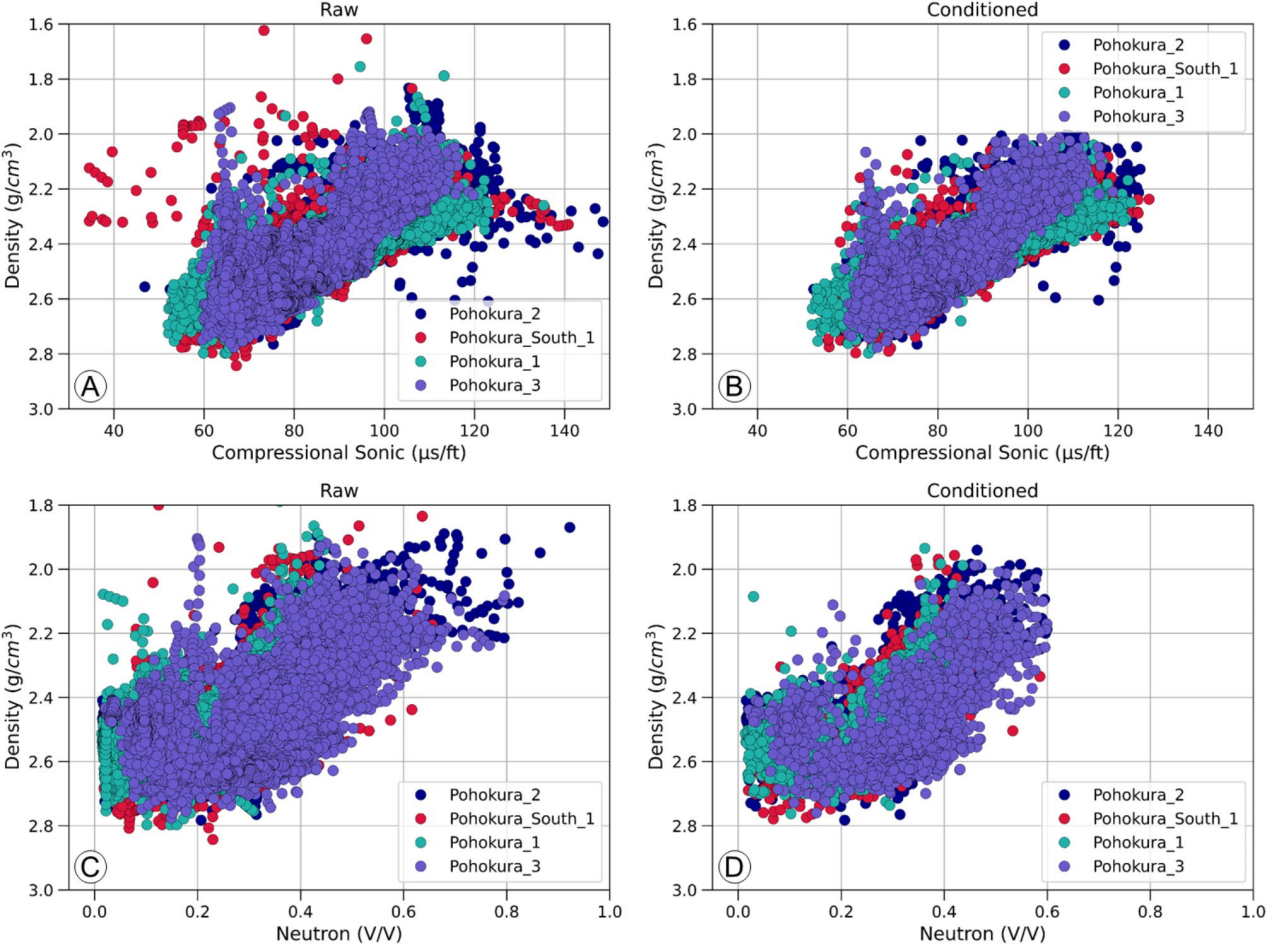
Crossplots between density and sonic (**A**,**B**), density and neutron porosity (**C**,**D**) showing the raw and conditioned data. Plots with conditioned data showing improved and consistent relationship.

There are several issues that can lead to inconsistent data during logging measurements in a borehole, leading to incorrect conclusions. These problems are particularly prevalent in sonic and density logs due to poorly washed-out intervals. In our case, the sonic logs showed sudden changes, known as spikes, due to the attenuation of the energy of the first-arrival wave signals^59^. The density logs also had a few erroneous intervals, causing a significant decrease in data quality. To address these problems, we used the de-spiking method, which significantly improved the data quality as shown in (Figs. 2 and 3) through a comparison of raw (Figs. 2A and B and 3A and B) and conditioned (Figs. 2C and D and 3C and D) data in histograms and crossplots. All the wells showed a consistent pattern, indicating improved data quality.

### Petrophysical analysis

In this research, we conducted a petrophysical analysis of four geophysical wells within the Mangahewa Formation in the Taranaki Basin. This included the measurement of porosity, permeability, volume of shale, types of shale and shale distributions, and fluid saturations for reservoir evaluation. In this study we utilized a series of well log data, namely caliper (CAL), gamma ray (GR), resistivity shallow (MSFL) and deep (ILD), density (DEN), neutron porosity (NPHI), compressional sonic (DTC), shear sonic (DTS), and spontaneous potential (SP) logs, to perform qualitative, quantitative, and model analysis. The employed data were extracted from the geophysical wells of Pohokura1 (P1), Pohokura2 (P2), Pohokura3 (P3), and Pohokura South1 (PS1) (Table 1).

**Table 1:** List of studied wells and available logs. Here, the availability and non-availability of the respective log data are denoted by Y and N, respectively.

Well name	CAL (inches)	GR (API)	Resistivity (Ωm)		DEN (g/cm^3^)	NPHI (v/v)	DTC (µs/ft)	DTS (µs/ft)	SP (mV)
			Shallow	Deep					
Pohokura1	Y	Y	N	Y	Y	Y	Y	N	Y
Pohokura2	Y	Y	Y	Y	Y	Y	Y	Y	Y
Pohokura3	Y	Y	Y	Y	Y	Y	Y	Y	Y
Pohokura South1	Y	Y	N	Y	Y	Y	Y	Y	N

The Python programming language was used for the various types of well log analysis and visualization including petrophysical and rock physics analysis. The essential geophysical well logs are de-spiked as well as removed error points/bad data and outliers by using OneClassSVM submodule of SVM (Support Vector Machine) module of unsupervised python machine learning techniques.

#### Volume of shale (Vsh) determination

The Eq. (1) was applied to estimate gamma ray index (I_GR_) to measure the shale volume^60^.

1 $${\text{IGR}}=\frac{{{\text{G}}{{\text{R}}_{{\text{log~}}}} - {\text{~G}}{{\text{R}}_{{\text{min}}}}}}{{{\text{G}}{{\text{R}}_{{\text{max}}}} - {\text{~G}}{{\text{R}}_{{\text{min}}}}}}$$

Where,

GR_log_ = gamma ray log value for the formation.

GR_max_, GR_min_ = maximum and minimum value of gamma ray log.

As the studied Mangahewa Formation of Kapuni Group in Taranaki Basin is of Eocene age, hence for the tertiary rock the function of Larionov model has been employed to reduce over estimation and calculate more accurate fraction or percent of volume of shale of the formation by using the following formula^61–63^.

2 $${\text{V}}_{{{\text{sh}}}} = {\text{ }}0.0{\text{83 *}}\left( {{\text{2}}^{{({\text{3}}.{\text{7 }}*{\text{ IGR}})}} {-}{\text{1}}} \right)$$

The volume of quartz is calculated by the following relation,

3 $${{\text{V}}_{{\text{quartz}}}}={\text{1}} - {{\text{V}}_{{\text{sh}}}}$$

Where, V_quartz_ = fraction of quartz volume.

V_sh_ = fraction of shale volume.

#### Density porosity

The Eq. (4) was applied to calculate density porosity from the density log.

4 $$\Phi _{{\text{D}}} = \frac{{\rho _{{{\text{ma}}}} - ~\rho _{{\text{b}}} }}{{\rho _{{{\text{ma}}}} - ~\rho _{{\text{f}}} }}$$

Where, Φ_D_ = fraction or percent of density porosity of the formation.

ρ_ma_ = matrix density of the formation, for sand 2.65 g/cm^3^.

ρ_b_ = bulk density of the formation from log.

ρ_f_ = fluid density, usually ranges from 1 to 1.1, but due to gas effect it becomes < 1 which results in high porosity.

#### Neutron porosity

The neutron porosity directly estimated from neutron porosity log responses and neutron porosity value is equivalent to neutron porosity log responses. The neutron porosity can be denoted as Φ_N_.

#### Sonic porosity

The Eq. (5) was applied for measuring the sonic porosity from the compressional sonic log^64^.

5 $$\Phi _{{\text{S}}}=\frac{{{\Delta\:}\text{t}}_{\text{l}\text{o}\text{g}}-\:{{\Delta\:}\text{t}}_{\text{m}\text{a}}}{{{\Delta\:}\text{t}}_{\text{f}}-\:{{\Delta\:}\text{t}}_{\text{m}\text{a}}}$$

Where, Φ_S_ = fraction or percent of sonic porosity of the formation.

Δt_log_ = interval transit time from log, (µs/ft).

Δt_ma_ = interval transit time of the matrix of the formation, for sandstone 54–56 (µs/ft).

Δt_f_ = interval transit time of fluids in the well bore, for mud filtrate 185–190 (µs/ft).

#### Total porosity and effective porosity

The density porosity and neutron porosity together are used to calculate total porosity by considering the presence or absence of gas in the formation by using the following equations^65,66^.

For gas bearing zone,

6 $$\Phi _{{\text{T}}}=\frac{{\varPhi\:}_{D}+\:{\varPhi\:}_{N}}{2}$$

For absence of gas in the formation,

7 $$\Phi _{{\text{T}}}=\sqrt{\frac{\left({{\varPhi\:}_{D}}^{2}+{{\varPhi\:}_{N}}^{2}\right)}{2}}$$

Where, Φ_T_ = total porosity or accurate porosity of the formation.

The following equation was used to estimate the effective porosity^67,68^.

8 $$\Phi _{{\text{e}}} = {\text{ }}\Phi _{{\text{T}}} *\left( {{\text{1}}{-}{\text{V}}_{{{\text{sh}}}} } \right)$$

#### Water saturation

The water saturation has been determined by two models namely the Archie’s model and the Indonesian model where the Archie’s model used for relatively clean sand reservoir and the Indonesian model used for relatively shaly sand reservoir for better results^69,70^.

The Eq. (9) was used to measure the water saturation (S_w_) for a clean sand unit based on the Archie’s model^71^.

9 $$S_{w} = \left( {\frac{{a*R_{w} }}{{\Phi ^{m} *R_{t} }}} \right)^{{1/n}}$$

Where,

a = formation tortuosity factor, 0.81 for consolidated sandstone, 0.62 for unconsolidated sandstone and 1 for shale.

R_w_ = water resistivity of the formation.

Φ = total porosity.

m = formation cementation exponent, for unconsolidated sandstone about 2.15 and for consolidated sandstone about 2.

R_t_ = formation true resistivity.

The Eq. (10) was used to measure the water saturation from a shaly unit based on the Indonesian model^72^.

10 $${\text{S}}_{{\text{w}}} = \left[ {\left\{ {\left( {\frac{{V_{{sh}}^{{2 - V_{{sh}} }} }}{{R_{{sh}} }}} \right)^{{1/2}} + \left( {\frac{{\Phi _{e}^{m} }}{{R_{w} }}} \right)^{{1/2}} } \right\}^{2} } \right]^{{ - 1/{\text{n}}}}$$

Where, S_w_ = water saturation.

Φ_e_ = effective porosity.

R_sh_ = shale resistivity.

V_sh_ = shale volume.

m = cementation coefficient.

n = saturation capacity.

R_w_ = formation water resistivity.

#### Permeability

The resistivity log and porosity logs are usually used for permeability estimation and porosity logs are more preferable than resistivity log because of high correlation between porosity and permeability^73^. The permeability of Mangahewa reservoir units was calculated based on irreducible water and porosity by Timur model with the help of the following equation^74,75^.

11 $$\:\text{K}=0.136\text{*}\frac{{\varPhi\:}^{4.4}}{{S}_{wir}^{2}}$$

Where, K = permeability (mD).

Φ = total porosity.

S_wir_ = Irreducible water saturation.

The Eq. (12) was used for calculating the irreducible water saturation.

12 $$\:\text{S}_{\text{w}\text{i}\text{r}}=\frac{\varPhi\:*\:{S}_{w}}{{\varPhi\:}_{e}}$$

### Rock physics analysis

The RPA contains the evaluation of petrophysical properties of a reservoir unit including porosity, fluid saturations, lithology based on elastic properties of the unit. The elastic properties of a rock vary with its compositions and textures.

#### Shear sonic velocity (Vs) prediction

To create a highly accurate RPM and RPT for a particular rock unit, it is necessary to have information on shear sonic velocity (Vs). In order to examine the sensitivity of the RPM and RPT, and to address the lack of available shear sonic log data, the prediction of shear sonic velocity is crucial. To do this, we first selected a training dataset and a test dataset. From the training dataset, we selected the features and target, while the test dataset only contained features that we needed to use to predict the target value. In this case, the shear sonic log was the target dataset, while the other logs were the features. Next, we conducted an exploratory data analysis on the training dataset, which included a seaborn pair plot and a heatmap correlation matrix. This helped us to understand the multivariate correlations and univariate frequencies as well as probability densities. It also helped us to identify any outliers in the dataset. We then transformed the dataset using StandardScaler and removed the outliers using OneClassSVM, a submodule of SVM (Support Vector Machine). This helped to make the distributions of the dataset more Gaussian and less skewed. We then used the machine learning algorithm GradientBoostingRegressor with hyperparameters to train the training dataset and build a model. Finally, we used this model to predict the targeted shear sonic value from the test features and denormalize this value to get the actual predicted value of the shear sonic log.

#### Matrix framework

Clastic rocks are typically made up of a combination of minerals, each of which has its own unique elastic modulus value. Consequently, the rock’s elastic modulus is the mean value derived from the average of the moduli of the individual minerals that make it up. In our research, we used the Voigt bounds, Reuss bounds, and the Voigt-Reuss-Hill average to estimate the elastic moduli of a rock matrix consisting of multiple minerals^29,30,76^. To determine the moduli for a rock with multiple minerals, we used the following two equations to calculate the Voigt upper bounds and Reuss lower bounds (Table 2).

13 $$\:{\text{M}}_{\text{v}\:}\:=\:\sum\:_{\text{i}=0}^{\text{N}}{\text{f}}_{\text{i}}{\text{M}}_{\text{i}}$$

14 $$\:\frac{1}{{\text{M}}_{\text{R}}}=\:\sum\:_{\text{i}=0}^{\text{N}}{\text{f}}_{\text{i}}\frac{1}{{\text{M}}_{\text{i}}}$$

Where, M_v_ = Voigt upper bounds.

M_R_ = Reuss lower bounds.

N = number of components of the rock.

f_i_ = i^th^ components volume.

M_i_ = i^th^ components elastic modulus.

The Voigt-Reus-Hill average (Hill average) is estimated by using the Eq.  (15),

15 $$\:{M}_{VRH}\:=\:\frac{{M}_{v}\:{+\:M}_{R}}{2}$$

#### Fluid properties and mixture

The elastic properties of a reservoir unit are greatly affected by pore fluids. In this study, an empirical model proposed by Batzle and Wang has been utilized to estimate the pore fluids properties of different types such as brine, oil and gas^77^.

As the Pohokura gas condensate field containing heterogenous fluids in mixture. The Wood’s average method employed to estimate the effective bulk modulus for the heterogenous fluids by using the following formula.

16 $$\:\frac{1}{{\text{K}}_{\text{f}}}\:=\:\frac{{\text{S}}_{\text{b}\text{r}\text{i}\text{n}\text{e}}}{{\text{K}}_{\text{b}\text{r}\text{i}\text{n}\text{e}}}+\:\frac{{\text{S}}_{\text{o}\text{i}\text{l}}}{{\text{K}}_{\text{o}\text{i}\text{l}}}+\:\frac{{\text{S}}_{\text{g}\text{a}\text{s}}}{{\text{K}}_{\text{g}\text{a}\text{s}}}$$

Where, K_f_ = effective bulk modulus of the fluid.

S_brine_, S_oil_, S_gas_ = saturation of brine, oil and gas, respectively.

K_brine_, K_oil_, K_gas_ = bulk modulus of brine, oil and gas, respectively.

#### Fluid substitution

Substituting different fluids in porous media can help us understand and predict how the fluids influence the elastic characteristics of the media, including acoustic impedance and seismic velocity, under reservoir conditions. The elastic properties of media at specific fluid saturation levels can be estimated by utilizing the following Gassmann equation. To do this, we need to have data on the velocity and density of the media, which can be used to calculate the initial parameters for the Gassmann equation by utilizing workflow described^39,78^. The initial calculations for the bulk and shear modulus involve using the V_P_, V_S_, and density data.

17 $$\:{K}_{sat1}\:=\:{\rho\:}_{1}\:({V}_{p1}^{2}-\:\frac{3}{4}{V}_{s1}^{2})$$

18 $$\:{\mu\:}_{sat1}\:=\:{\rho\:}_{1}{V}_{s1}$$

To calculate bulk modulus at various fluid saturation conditions, the following Gassmann’s equation has been applied.

19 $$\:\frac{{K}_{sat2}}{{K}_{s}-\:{K}_{sat2}}-\:\frac{{K}_{fl2}}{{\varPhi\:(K}_{s}-\:{K}_{fl2})}=\:\frac{{K}_{sat1}}{{K}_{s}-\:{K}_{sat1}}-\:\frac{{K}_{fl1}}{{\varPhi\:(K}_{s}-\:{K}_{fl1})}$$

Where,

$\:{K}_{sat1}$ and $\:{K}_{sat2}$ = saturated rock bulk moduli for fluid1 and fluid2, respectively.

$\:{K}_{fl1}$ and $\:{K}_{fl2}$ = bulk moduli of the fluid1 and fluid2, respectively.

As fluid does not have any resistance to prevent shear deformation, the effective shear moduli will be the same for those saturated conditions.

$$\:{\mu\:}_{sat1}=\:{\mu\:}_{sat2}$$

The rectified bulk density for the fluid was estimated by utilizing the equation below,

$$\:{\rho\:}_{sat2}=\:{\rho\:}_{sat1}+\:{\varPhi\:(\rho\:}_{fl2}-\:{\rho\:}_{fl1})$$

Now, for the new fluids the new velocities are calculated by the following Eq. 

20 $$\:{V}_{psat2}=\:\sqrt{\frac{{K}_{sat2}+\:\frac{4}{3}{\mu\:}_{sat2}}{{\rho\:}_{sat2}}}$$

21 $$\:{V}_{ssat2}=\:\sqrt{\frac{{\mu\:}_{sat2}}{{\rho\:}_{sat2}}}$$

#### Elastic framework

An effective rock framework can be created using the velocity versus porosity cross plot as a tool. The stiffness and softness of a rock were determined by utilizing the effective Voigt and Reuss elastic bounds. The Hashin-Shtrikman^79^ bounds are a helpful and effective way to describe the combination of elastic properties in a rock. The Hertz-Mindlin^26,80^ contact theory has been applied to compute the bulk and shear moduli of dry rock under significant porosity conditions.

22 $$\:{K}_{HM}=\:\sqrt[3]{\frac{{n}^{2}\:(1-{\varPhi\:}_{c}{)}^{2}{\mu\:}_{m}^{2}P}{18\pi\:(1-v{)}^{2}}}$$

23 $$\:{\mu\:}_{HM}=\:\frac{5-4v}{5(2-v)}\sqrt[3]{\frac{{3n}^{2}\:(1-{\varPhi\:}_{c}{)}^{2}{\mu\:}_{m}^{2}P}{2{\pi\:}^{2}(1-v{)}^{2}}}$$

Where, K_HM,_ µ_HM_ = dry rock bulk and shear moduli based on Hertz – Mindlin contact theory.

P = effective confining pressure.

v = Poisson’s ratio.

n = coordination number.

Φ_C_ = critical porosity.

24 $$\:{\text{K}}_{\text{d}\text{r}\text{y}}=[\frac{\frac{{\Phi\:}}{{{\Phi\:}}_{\text{c}}}}{{\text{K}}_{\text{H}\text{M}}+\:\frac{4}{3}{{\upmu\:}}_{\text{H}\text{M}}}+\:\frac{1-\:\frac{{\Phi\:}}{{{\Phi\:}}_{\text{c}}}}{\text{K}+\:\frac{4}{3}{{\upmu\:}}_{\text{H}\text{M}}}{]}^{-1}-\:\frac{4}{3}{{\upmu\:}}_{\text{H}\text{M}}$$

25 $$\:{{\upmu\:}}_{\text{d}\text{r}\text{y}}=[\frac{\frac{{\Phi\:}}{{{\Phi\:}}_{\text{c}}}}{{\text{K}}_{\text{H}\text{M}}+\:\text{z}}+\:\frac{1-\:\frac{{\Phi\:}}{{{\Phi\:}}_{\text{c}}}}{{\upmu\:}+\:\text{z}}{]}^{-1}-\:\text{z}$$

26 $$\:\text{z}=\:\frac{{{\upmu\:}}_{\text{H}\text{M}}}{6}\left(\frac{9{\text{K}}_{\text{H}\text{M}}+8{{\upmu\:}}_{\text{H}\text{M}}}{{\text{K}}_{\text{H}\text{M}}+\:{2{\upmu\:}}_{\text{H}\text{M}}}\right)$$

Where, $\:{K}_{dry}$ and $\:{\mu\:}_{dry}$ = bulk and shear moduli of unconsolidated sand.

K = bulk modulus of the mineral.

Φ = porosity.

**Table 2:** Table of symbols.

Symbol	Definition
V_P_	Compressional sonic velocity
V_S_	Shear sonic velocity
IP	Acoustic impedance
RPA	Rock physics analyses
RPM	Rock physics models
RPTs	Rock physics templates
GPa	Gigapascal
K	Permeability
Φ, Φ_C,_ Φ_e,_ Φ_S,_ Φ_D,_ Φ_T_	Porosity, critical porosity, effective porosity, sonic porosity, density porosity, total porosity
K, µ	Bulk modulus, shear modulus
VRH	Voigt-Reus-Hill average
K_HM_ and µ_HM_	Dry rock bulk and Shear moduli based on Hertz–Mindlin contact theory
$\:{K}_{dry}$ and $\:{\mu\:}_{dry}$	Bulk and shear moduli of unconsolidated sand.
v	Poisson’s ratio
n	Coordination number, saturation capacity
P	Effective confining pressure
S, S_w_, S_wir_	Saturation, water saturation, irreducible water saturation
ρ	Density
M_v_	Voigt upper bounds
M_R_	Reuss lower bounds
R_w_, R_t_	Formation water resistivity, formation true resistivity
I_GR_	Gamma ray index
V_sh_, V_quartz_	Volume of shale, volume of quartz
m	Cementation coefficient

## Results and discussion

### Petrophysical analysis

Various methods were utilized to examine the P1, P2, P3, and PS1 well data for assessing the hydrocarbon potential and the quality of reservoir in the Mangahewa Formation. Both quantitative and qualitative methods were utilized, including crossplots of different well log measurements to analyze lithology, fluid type, and saturation. The shale volume (Vsh), a crucial indicator of reservoir quality, was determined through the gamma log, and porosity calculations were based on the density log. If the density log was unreliable, sonic porosity was used instead. The neutron porosity and density porosity average was used to analyze gas zones.

#### Qualitative analysis

A variety of cross-plots were created using different well logs to determine the mineralogical and lithological makeup of the Mangahewa Formation. These plots include the combination of gamma-ray and neutron porosity data, neutron porosity and density data, neutron porosity and sonic data, and M-N data.

**Fig. 4:**
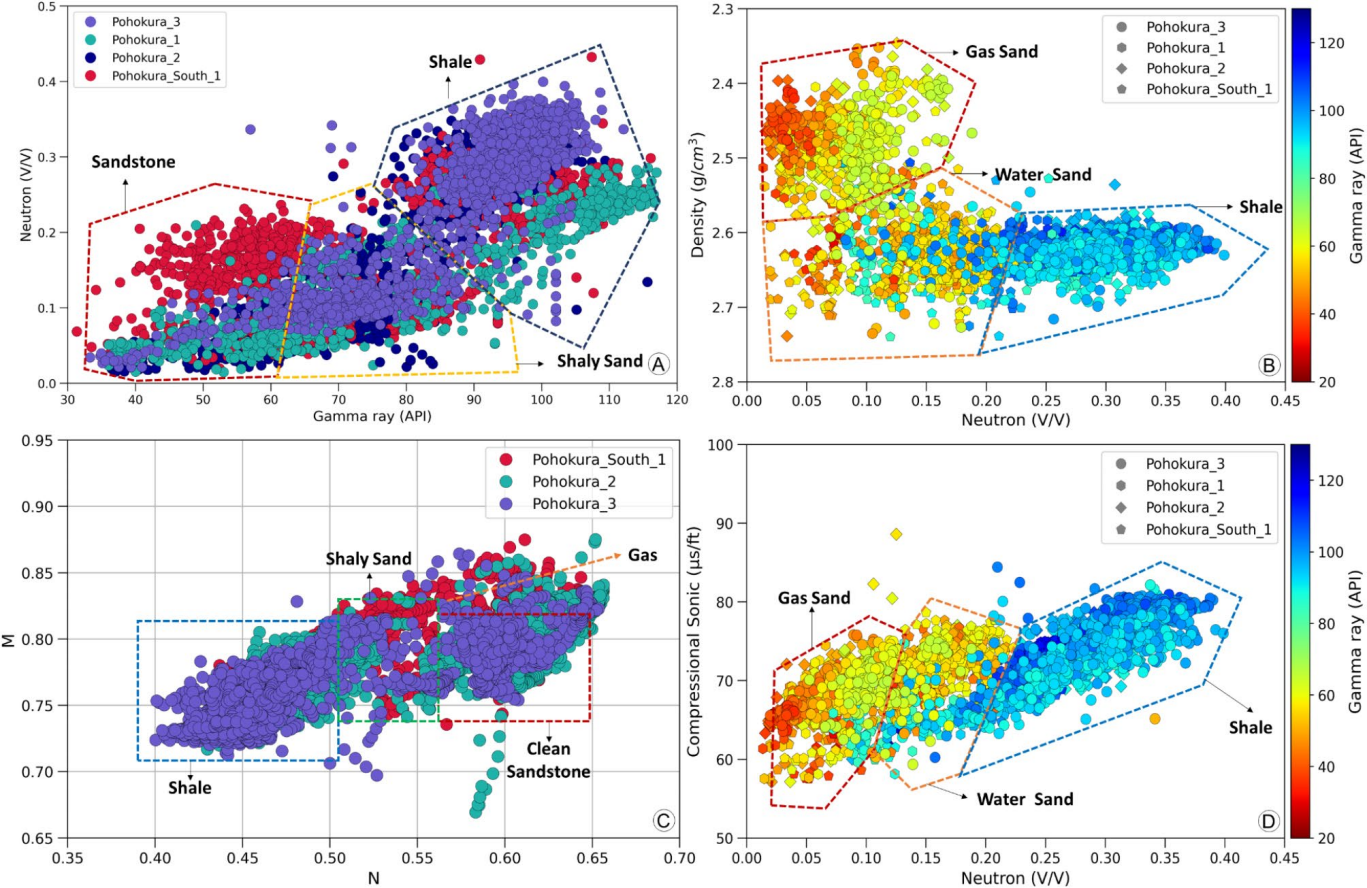
Crossplots illustrating lithological and fluid characteristics across four wells, highlighting sandstone and shale as dominant lithologies and gas and water as primary fluids. (**A**) Crossplot of gamma ray versus neutron porosity, where low gamma ray and neutron porosity values indicate sandstone, and high values suggest shale presence. (**B**) Density versus neutron porosity crossplot, showing gas presence in the reservoir, evident from low readings in both density and neutron porosity. (**C**) M-N plot where high values indicate sandstone. (**D**) Sonic versus neutron porosity crossplot further corroborates gas presence within the reservoir.

**Fig. 5:**
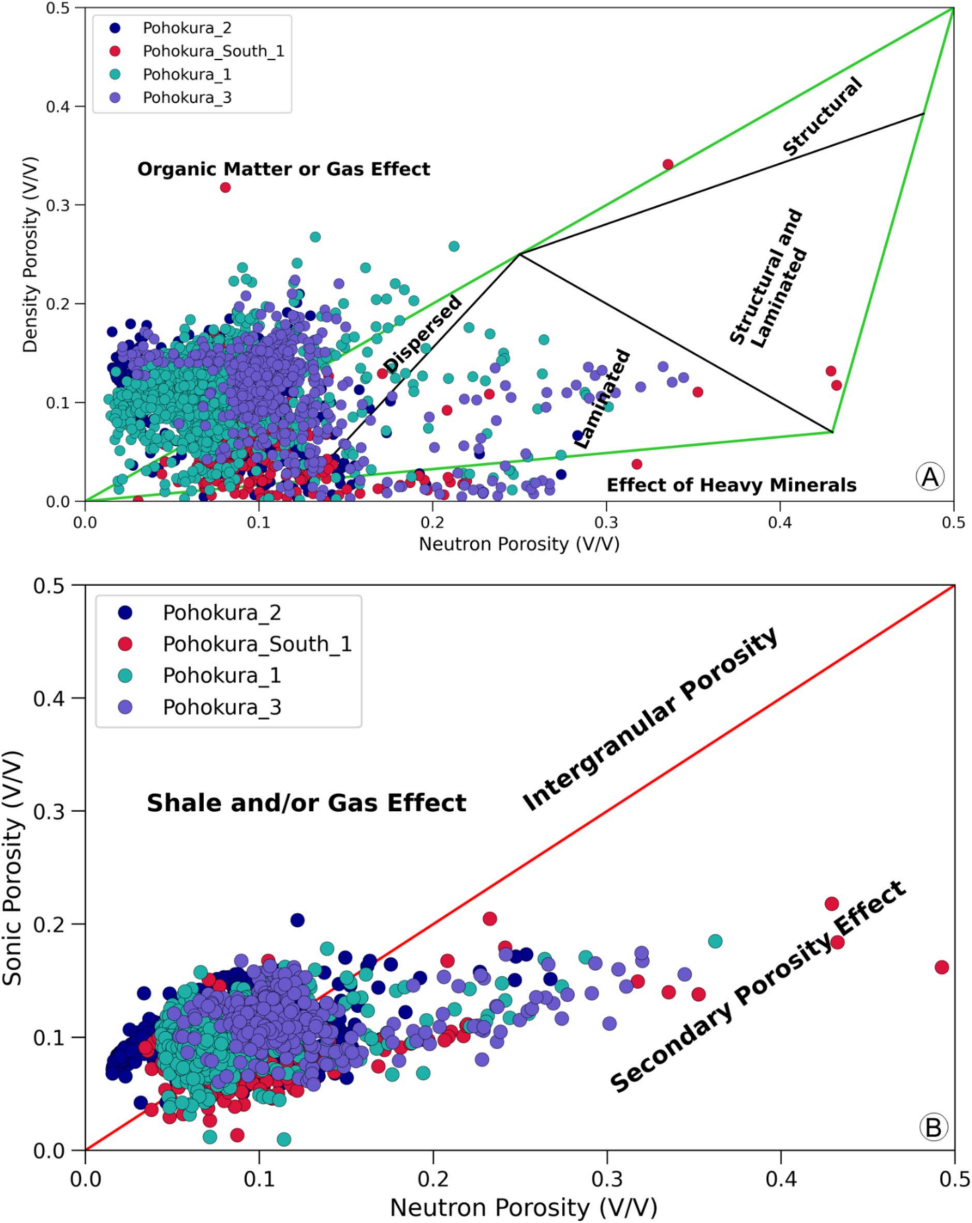
Crossplots illustrating reservoir characteristics: (**A**) Density vs. neutron porosity, showing dispersed shale distribution throughout the reservoir; (**B**) Sonic vs. neutron porosity, indicating that porosity is predominantly intergranular.

Three identifiable clusters emerge from the gamma-ray versus neutron porosity plot data: clean sand is characterized by low values in both gamma-ray and neutron porosity, dirty sand is represented by high values in both, and shale is indicated by elevated values in both neutron porosity and gamma-ray (Fig. 4A). Overall, the plots suggest that the Mangahewa Formation is made up of high-quality sandstone with interspersed shale. The observations derived from the cross-plot of neutron porosity log against density log incorporating gamma ray as the third axis are consistent with this interpretation. Points with low gamma ray-neutron porosity-density values correspond to gas sand, while points with high density and neutron porosity values, coupled with low gamma ray readings, are indicative of water sand (Fig. 4B). The cross-plot of neutron porosity log against sonic log with gamma ray in the third axis also exhibits similar characteristics (Fig. 4D). Gas is identified as the primary fluid in the reservoirs, with the majority of data points in the P1, P2, P3, and PS1 wells indicating sandstone as the predominant lithology in the Mangahewa Formation, as revealed by the M-N cross-plot (Fig. 4C). In addition, the shale distribution type was identified through different plots, which is important in determining hydrocarbon producibility. Examination of the cross-plot between neutron porosity and density porosity (Fig. 5A) revealed a predominantly dispersed distribution of shale, with occasional small clusters exhibiting laminated characteristics. The upward trend observed in certain data points may be attributed to the influence of gas or organic matter within the Farewell Formation. The effective porosity of a sample is adversely affected by dispersed clay minerals, and if left uncorrected, this could have implications for fluid saturation in the reservoir. Neutron porosity and sonic porosity cross-plot (Fig. 5B) shows both primary and secondary porosity is common in this reservoir.

#### Quantitative analysis

In all four studied wells (P1, P2, P3, and PS1), the Mangahewa Formation was found at similar depths. The thickness of this formation varied across the wells, with values of 381, 226, 280, and 333 m, respectively. To determine water saturation, the Indonesian equation was used, and gas saturation was calculated based on the water saturation estimate. A threshold of 50% Vsh was utilized to differentiate between shale and sand. Total porosity was calculated using density log data and then adjusted for gas and faulty measurements. A cut-off value of 9% porosity was used to identify reservoir zones. Table 3 exhibits the total and effective porosity in the wells under study. A 50% cut-off was applied for water saturation to separate the hydrocarbon saturated net pay zones and water saturated non-pay zones. Altogether, a total of 14 net pay intervals were recognized across the four wells. The PS1 well had the most pay zones (5), while the P1 well had the fewest (2). Table 3 displays the pay zones along with their corresponding petrophysical parameters.

**Table 3:** Summary of the petrophysical properties of the Mangahewa reservoir sands in different fields.

Well name	Thickness (m)					Porosity (%)		Shale (%)	Fluids (%)
	Top	Bottom	GT	NST	NRT	Φ_T_	Φ_e_	V_sh_	S_w_
Pohokura1	3520	4210	690	526	479	20.2	15.4	23.7	27
Pohokura2	3580	3810	230	172	157	19.6	14.7	25.2	24.2
Pohokura3	3530	3810	280	220	200	20.7	16.2	21.6	34.3
Pohokura South1	3550	3800	250	180	164	17.5	12.6	27.8	22

### Rock physics analysis

#### Shear Sonic prediction

**Fig. 6:**
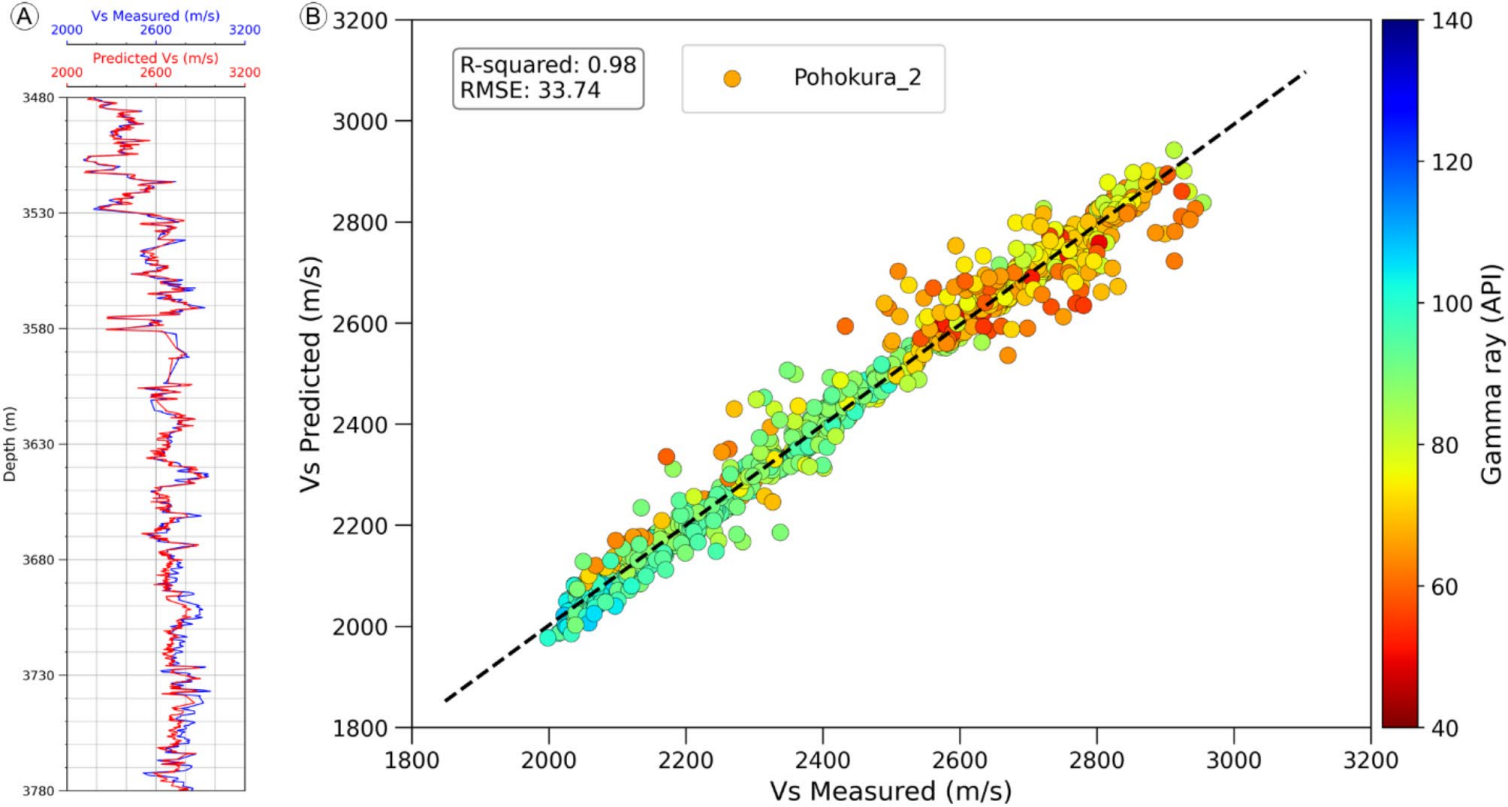
Comparison of measured and predicted shear sonic velocities in the Pohokura-2 well. (**A**) Log plot showing alignment between measured and predicted values. (**B**) Correlation plot with a 98% match, confirming strong predictive accuracy.

The shear sonic velocity (V_S_) prediction after data conditioning and petrophysical analysis is a vital task for rock physics analysis. In Fig. 6, the predicted shear sonic velocity (V_S_ Predicted) for P2 well is predicted by using various libraries of python data science and machine learning algorithms. The training datasets encompass data from the PS1 and P3 fields, whereas the test dataset is derived from the P2 field. The features include Gamma Ray, Density, Neutron porosity, photoelectric factor (PEF), Resistivity, and compressional sonic log (DTC), while the target variable is the shear sonic log (DTS). The train datasets are used for building a model and by using this model, from the test features of P2 predict the targeted shear sonic log. From this prediction of shear sonic log observed that the predicted shear sonic velocity (V_S_ Predicted) is 98% linearly correlated with the measured shear sonic velocity (V_S_ measured). The predicted shear sonic velocity (V_S_ Predicted) for P2 well is consistent, showing uniformity and high positive correlation value almost 98% with measured shear sonic velocity (V_S_ measured) (Fig. 6).

#### Data quality checking

Within the field of rock physics, verifying the accuracy of elastic logs is crucial, and this is accomplished through a procedure known as quality control. A method to achieve this involves establishing a connection between reservoir and elastic properties through the utilization of a tool known as a cross plot. This tool is commonly used in RPA for understanding the reservoir condition by plotting V_S_ against V_P_ or density against V_P_. The V_P_-V_S_ and V_P_-density domains are particularly useful for identifying different rock facies and understanding the composition of the rock. However, it can be challenging to differentiate between rock facies based on compressional sonic velocity alone, as overlapping velocities are common in deeper reservoirs. By adding shear sonic velocity (V_S_) to the equation, it becomes easier to understand the composition of each rock facies and the uncertainty surrounding their separation.

**Fig. 7:**
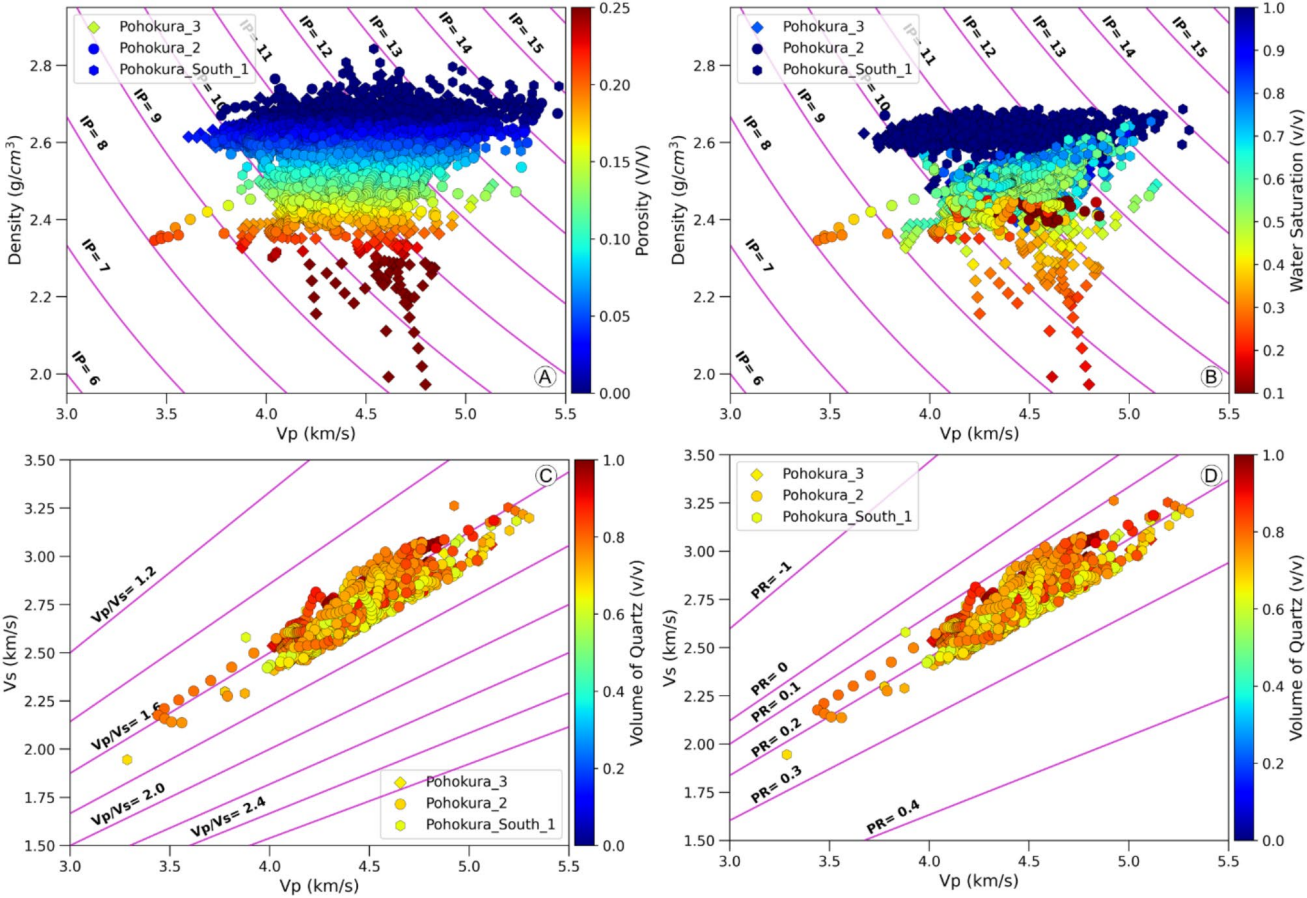
Plots of compressional sonic velocity (V*p*) versus density, color-coded by porosity (**A**) and water saturation (**B**). Plots of compressional sonic versus shear sonic, color-coded by quartz volume (**C**,**D**). Poisson’s ratio (PR) and acoustic impedance (IP) are also indicated.

In two cross plots, data from the P3, P2, and PS1 well show a trend of a superimposed poisson’s ratio (PR) line with a value of 0.2 and a V_P_/V_S_ ratio line with a value of 1.6, indicating that the reservoir may be composed of monomineralic sandstone (Fig. 7C, D). The volume of quartz color index also supports this interpretation. The cross plot between density and compressional sonic velocity (V_P_) can provide information about lithology, fluid saturation, and porosity (Fig. 7A, B). Decreased density and velocity are linked to a lower acoustic impedance, often indicating higher porosity and hydrocarbon saturation. Conversely, elevated acoustic impedance is frequently linked to rigid sand, where the impact of fluid saturation on both density and velocity is less pronounced compared to the influence of porosity. In this case, it may be difficult to differentiate between fluid saturations. In one of the plots, the hydrocarbon zone is distributed in both lower and higher acoustic impedance bearing zones, with the highest density and acoustic impedance representing shale. The shale displays the least porosity and the elevated water saturation, causing the density log to be more sensitive at deeper depths than the compressional sonic log.

The crossplot of IP and V_P_/V_S_ serves as an effective method for determining the type of rock, its quality, and its porosity and saturation. Typically, the existence of hydrocarbons is indicated by a reduced V_P_/V_S_ ratio and lower IP. As illustrated in Fig. 7A,B, the impact of fluid saturation on acoustic impedance is more pronounced in high porosity regions and less significant in low porosity areas within the V_P_/V_S_ domain. The IP versus V_P_/V_S_ domain clearly distinguishes between high porosity and low porosity zones at an acoustic impedance value of 11,600 (m/s * g/cm^3^) (Fig. 7A,B).

#### Mineral modelling

**Fig. 8:**
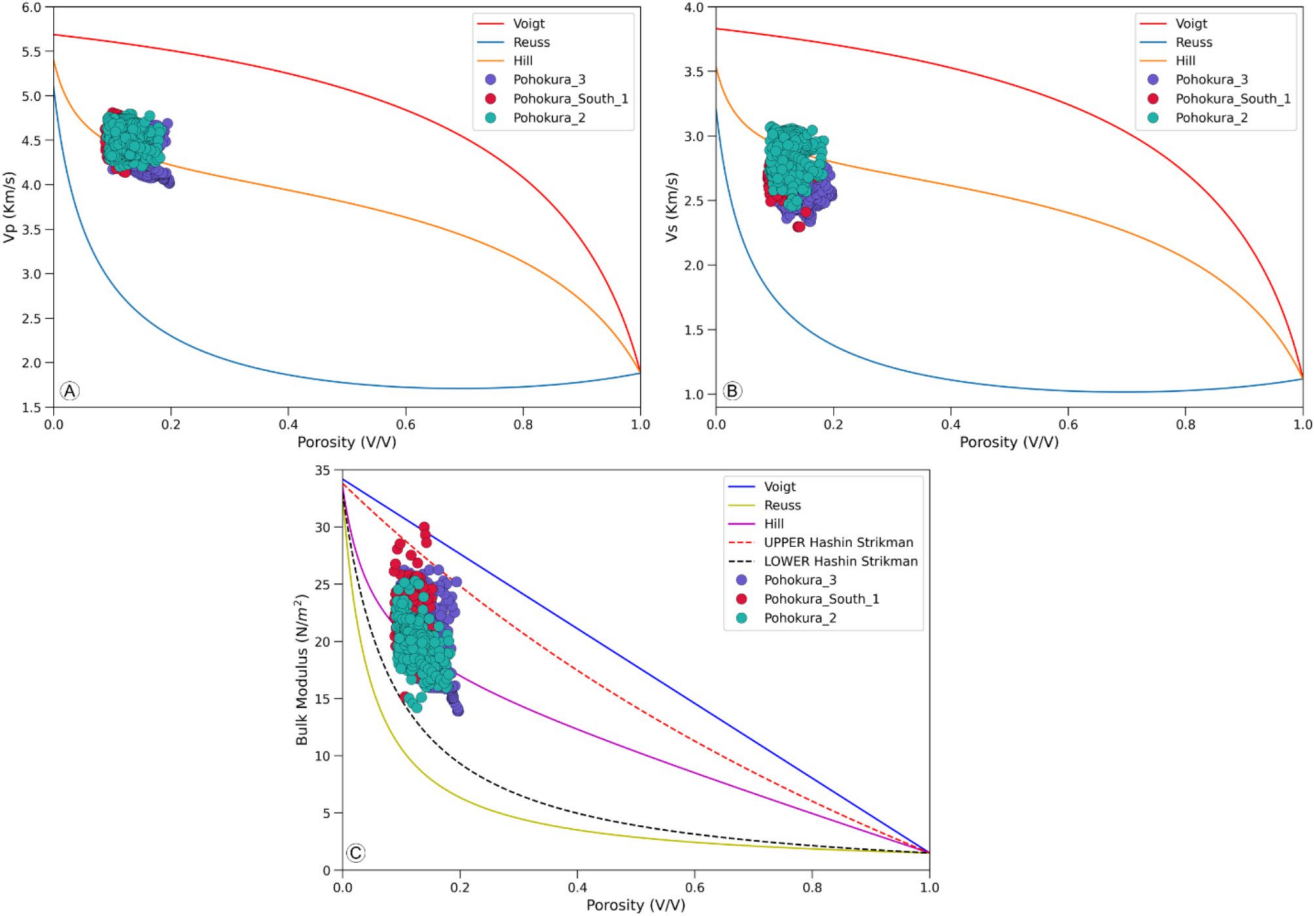
Range of critical porosities (0.1 to 1) modeled by the Voigt-Reuss bounds, alongside the Hashin-Shtrikman upper and lower limits.

Thin section analysis shows that the reservoir’s matrix comprises both sand and shale. To examine the physical constraints of different mineral combinations and ensure data quality, the elastic bounds of effective elastic moduli are utilized. Figure 8 illustrates the Voigt and Reuss effective elastic bounds, representing the theoretical upper and lower bounds of moduli for solid and fluid endpoints, respectively.

The arithmetic average of these two effective moduli is represented by Hill. Moreover, the modified Hashin-Shtrikman bounds, within a porosity range of 0.1 to 1, differentiate between the trends of unconsolidated and consolidated elastic moduli based on the critical porosity of the rock, providing insights into its diagenetic history (Fig. 8C). As cementation increases, porosity typically decreases. In this scenario, the critical porosity value for all the reservoir units is 0.4. The elastic moduli mentioned are contingent on the elastic characteristics of the constituents comprising the rock. Scattered data points in Fig. 8C indicate that the rock has a higher volumetric stiffness, as evidenced by its higher bulk modulus in the range of 15–30 N/m^2^. Additionally, the rock’s stiffness is evident in the higher V_P_ in the range of 4000–4900 m/s (Fig. 8A) and V_S_ in the range of 2400–3200 m/s (Fig. 8B).

#### Fluid modelling

Temperature, pressure, and dissolved constituents affect the elastic properties of water, specially density and elastic moduli. The properties of gases and oils are less stable and can fluctuate depending on their composition and temperature^81^. By using the Batzle and Wang method^77^, fluid properties were recalculated under reservoir conditions. The elastic properties of fluids input and output are shown in (Table 4). Small quantities of gas can exert a significant impact on the elastic properties of fluid systems. The existence of gas attenuates the fluid system’s P-impedance sharply in this case.

#### Fluid substitution analysis

**Fig. 9:**
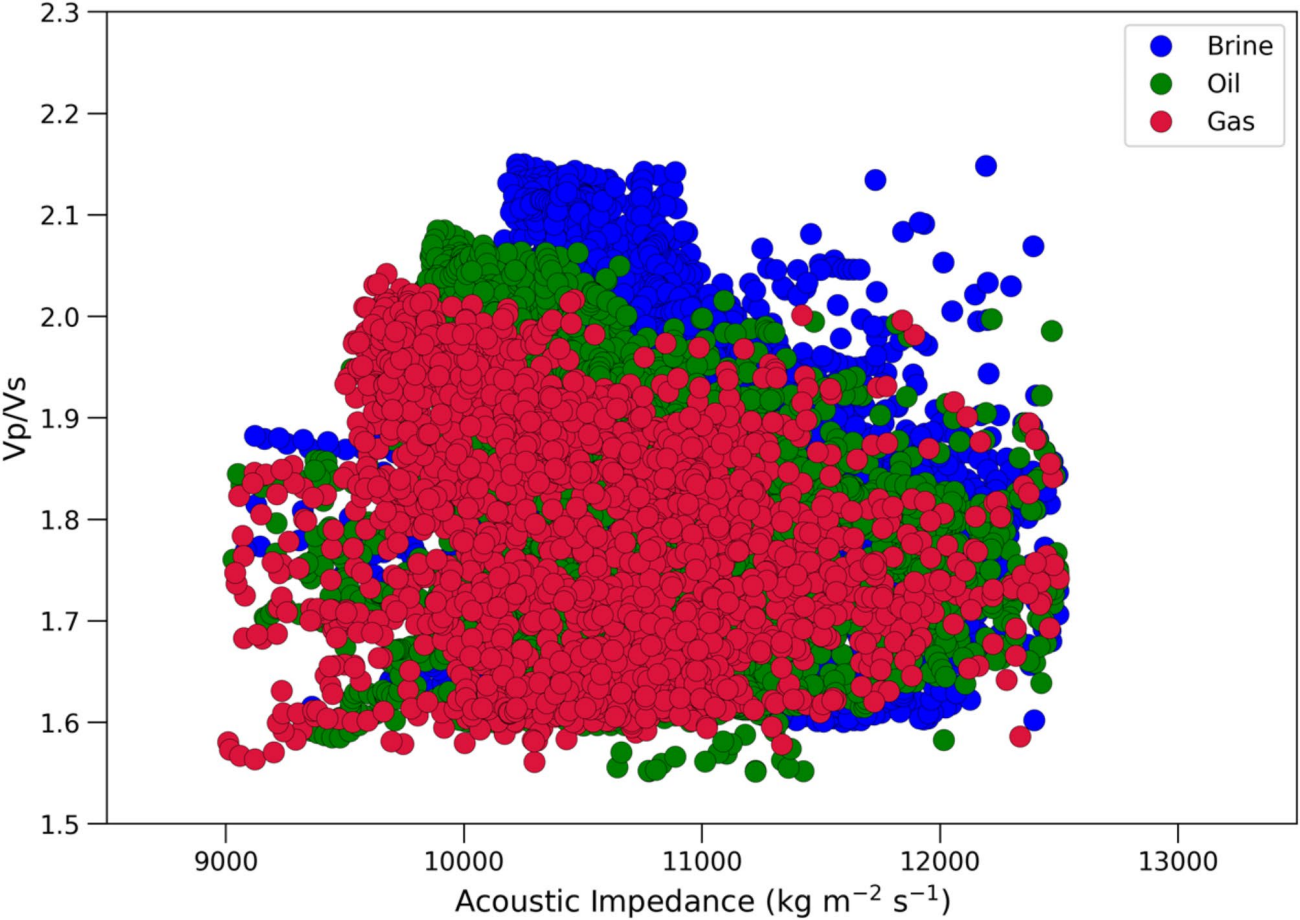
Cross-plot of acoustic impedance (IP) versus Vp/Vs for all wells, showing data under three distinct scenarios.

To test the presence of gas, oil and water within a formation, Gassmann’s model has been applied to three sets of observations after determining the rock matrix and fluid properties and predicted shear sonic velocities (V_S_). The three (3) scenarios considered for Gassmann fluid substitution analysis are 100% brine for water saturated reservoir, 20% brine and 80% oil for oil saturated reservoir and 10 brine and 90% gas for gas saturated reservoir. In Fig. 9, the three clusters of red, green, and blue colors depict reservoir sand saturated with gas, oil, and brine. This representation is achieved through Gassmann’s model for fluid substitution, considering the three distinct scenarios mentioned. Figure 9 shows that gas saturated sands lie between 1.6 and 2 in V_P_/V_S_ and 9000 to 12,000 in acoustic impedance value. The lower IP indicates the higher porosity and the lower V_P_/V_S_ ratio indicates the hydrocarbon saturation. This makes the V_P_/V_S_ ratio an effective tool for separating fluids.

#### Rock physics modelling

**Fig. 10:**
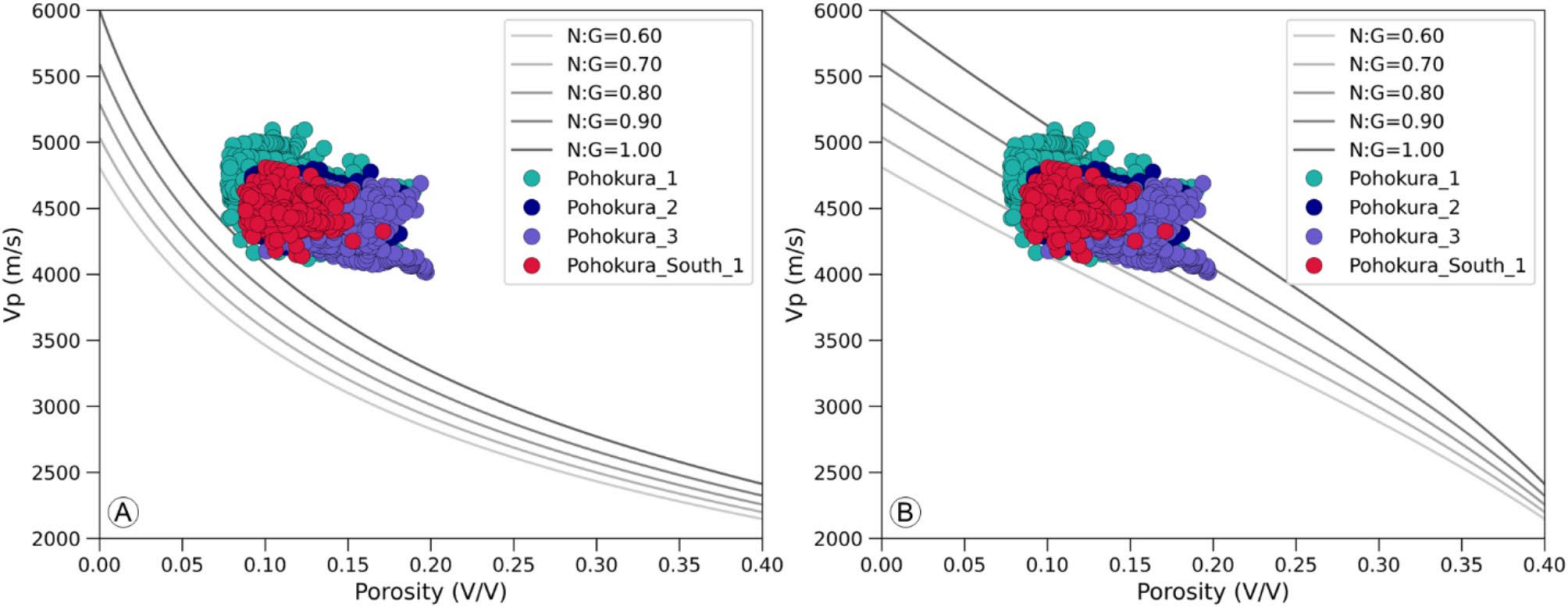
Crossplot of compressional sonic velocity and porosity showing that stiff sand model fits the data (**A**) and the net to gross is about 80% (**B**).

In the context of rock physics modeling, model lines for soft sand and stiff sand were overlaid on the cross-plot of compressional sonic velocity (V_P_) against porosity (Fig. 10) for the reservoir sands of P1, P2, P3 and PS1 wells. The cross plots (Fig. 10A,B) reveals that the plotted datapoints matched with the stiff sand model and the net to gross (NG) ratio falls between 70 and 95% for most of wells. Although the values of NG ratios for the reservoir sands are higher across all the wells, the porosity is lower in the range of 8–19% for all the wells which results due to the stiff behavior of the reservoir sands. The compressional sonic velocity (V_P_) ranges between 4100 and 5000 m/s and the combination of lower porosity and higher NG sand ratios in all the wells suggests an increased density of the reservoir unit as well as the greater compaction of the unit.

#### Rock physics template

**Fig. 11:**
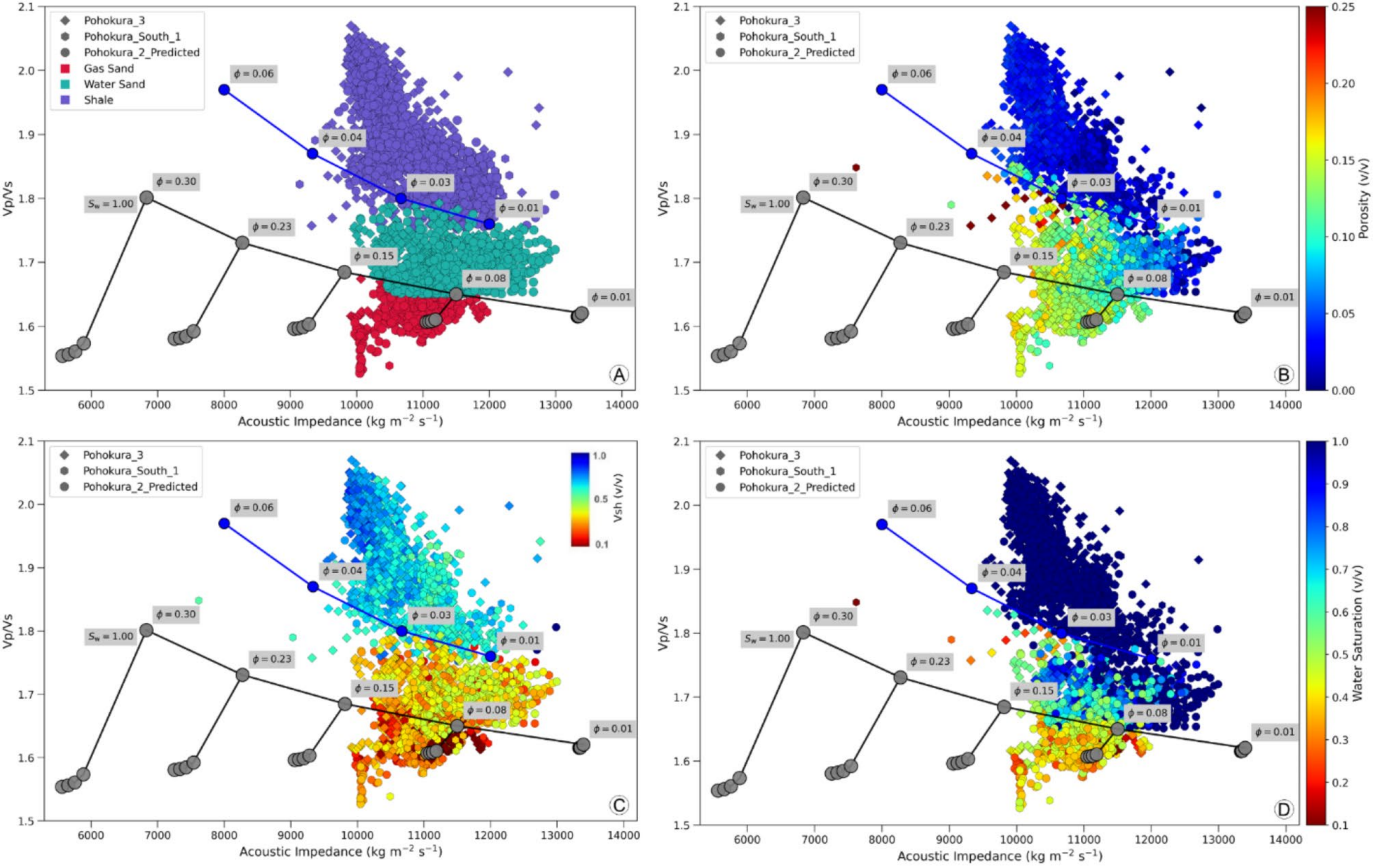
The calibrated model demonstrates the accuracy of the stiff sand RPT model in distinguishing between different lithologies and fluid types. (**A**) Facies distribution, (**B**) Porosity, (**C**) Volume of shale, (**D**) Water saturation.

**Fig. 12:**
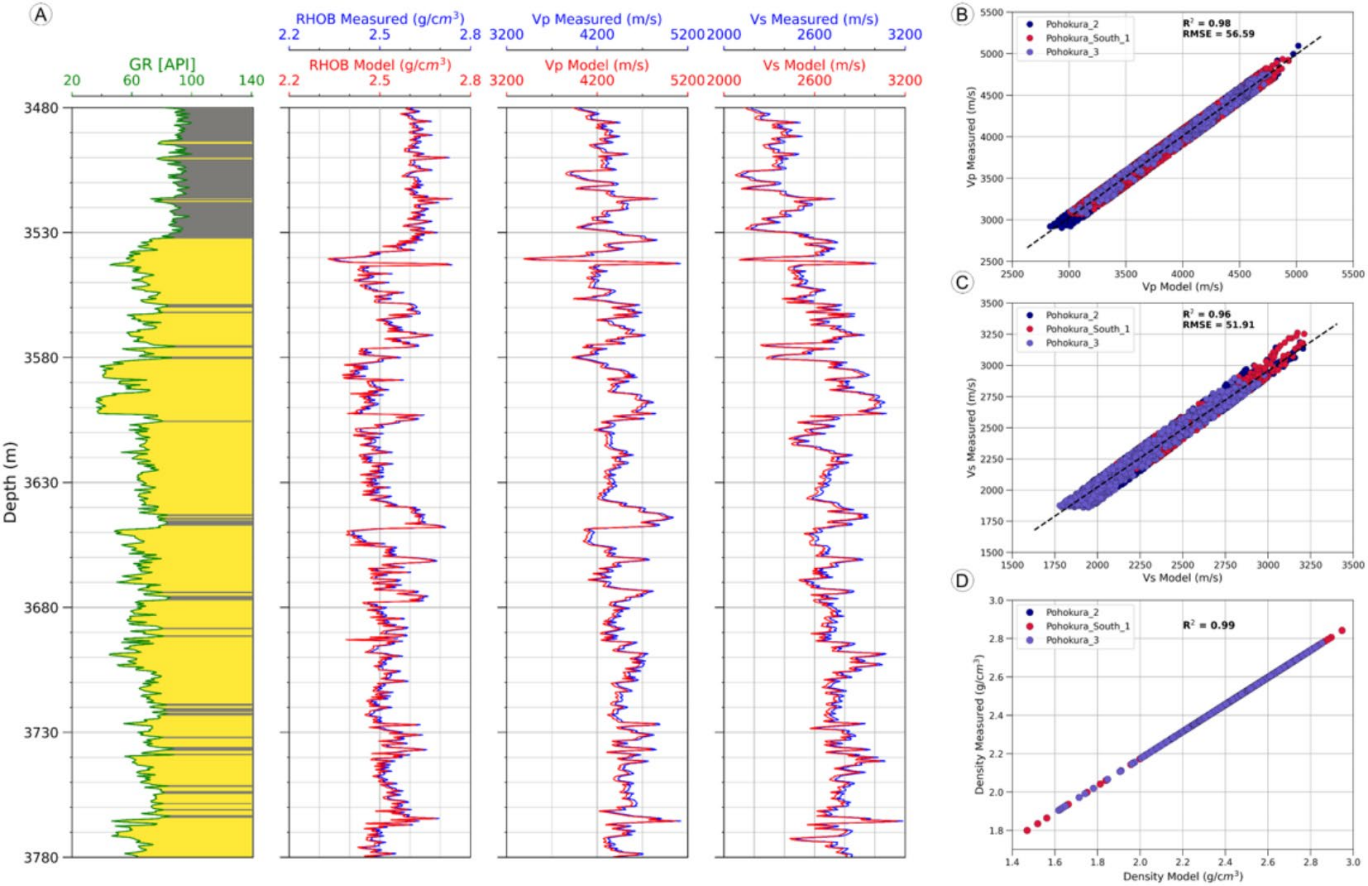
Comparison of measured and modeled data across all wells (**A**–**D**) for lithology, density, compressional sonic, and shear sonic properties in Pohokura 2. The results show strong correlation between the measured and modeled values.

The RPTs were first elucidated by Avseth and Odegard^82^ employing the framework established by Dvorkin et al.^27^. Subsequently, Avseth et al.^39^ endorsed them as a valuable tool or model for understanding the elastic characteristics of reservoirs, considering variations in mineral compositions, porosity, fluid types, and saturations. The Depositional model, Diagenetic Trend model and Gassmann’s Fluid Substitution model together develop rock physics models and rock physics templates^39^. The model incorporated a variety of porosity and saturation variations to assess the alterations in reservoir behavior. The V_P_, V_S_ and density of the model and measured data are matched very well after calibrating the developed RPM by borehole geophysical data, which reveals that a proper model is developed and it can be effective for further analysis. The rock physics models and templates develop dry-rock structure with shear and bulk modulus and these two moduli illustrate the capacity of the structure to bear tangential and normal stress, respectively. The Gassmann’s equation is applied to estimate the moduli of both dry and saturated rock. The V_P_ and the V_S_ for the saturated rock measured by the calculated saturated rock moduli and density. The IP is determined by multiplying the V_P_ and density. A predicted well (P2_Predicted) is developed by following the above procedures with locally constrained different shale volume, porosity, water saturation, mineralogy and rocks and fluid properties. The RPTs illustrate the cross plot between the ratio V_P_/V_S_ and IP, with porosity and saturation lines (Fig. 11A–D). The porosity for sand ranges from 1 to 30% with 7% increments denoted by dark gray line and for shale ranges from 1 to 6% with 2% increments denoted by dark blue line in the RPT of this study (Fig. 11). The data of PS1 and P3 for depths ranging from 3550 to 3800 m and 3530 m to 3810 m, respectively, pertain to the Mangahewa Formation. These are plotted as a cross plot in the RPT (Fig. 11). The cross plot reveals the separation among different facies, namely shale facies, water sand facies, and gas sand facies. The porosity of gas sand varies from 7 to 23%, and the gas saturation ranges from 66 to 88%. The porosity of water sand and shale ranging from 3 to 12 and 1–5%, respectively. The RPT initially generated for PS1 and P3 wells and then tested by the data of P2_Predicted well. The data of P2_Predicted well at a depth range 3580 m to 3810 m of Mangahewa Formation is superimposed on the cross plots of PS1and P3 in the RPT (Fig. 11). The cross plot of the tested well also reveals demarcation among different facies, encompassing the shale facies, the water sand facies and the gas sand facies. The lithology and fluid content identified in the tested well (P2_Predicted) is completely correlated with lithology and fluid content identified from the other measured geophysical logs (Fig. 12).

As the reservoir zone for all the wells located at higher depth in the range of 3200 to 4000 m. Therefore, higher burial pressure and diagenetic changes result nearly same acoustic impedance value for all rock facies including shale facies, water sand facies and gas sand facies. In such case, only acoustic impedance failed to separate these facies from each other. Other hand, V_P_/V_S_ ratios have significant sensitivity to separate shale facies, water sand facies and gas sand facies. In Fig. 9, the gas sand facies, the water sand facies and shale facies spanning within the range of 1.52 to 1.65, 1.65 to 1.75, 1.75 to 2.05 V_P_/V_S_ ratios, respectively.

The changing pattern of porosity, saturation and shale content closely matched with the changing pattern of measured porosity, saturation and volume shale content which reflects the sensitivity of the RPT, as presented in (Fig. 11).

**Fig. 13:**
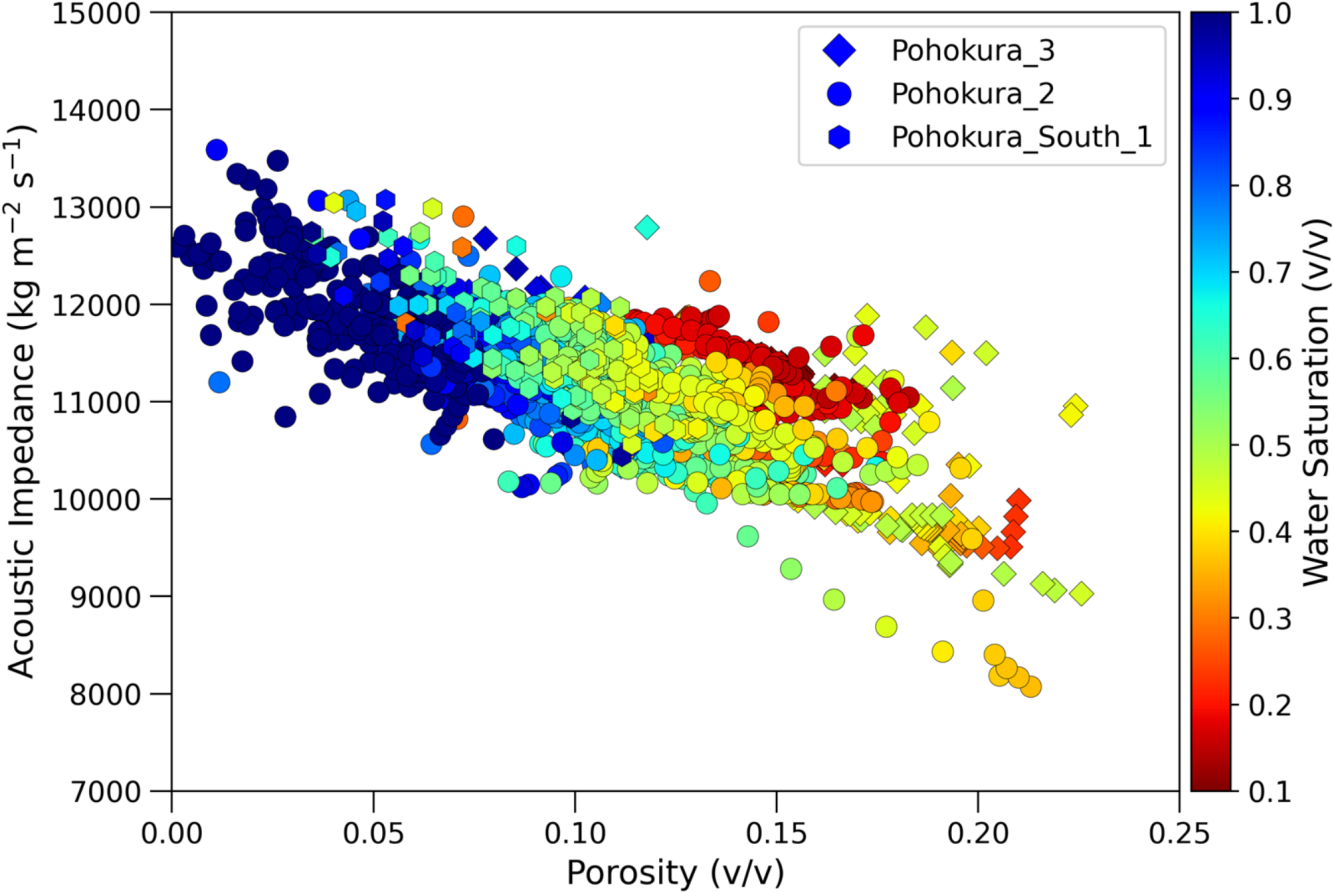
Crossplot of acoustic impedance (IP) versus porosity, demonstrating a strong correlation, which indicates that porosity can be estimated from seismically derived acoustic impedance.

The reliability of the predictive model implies that the foundation of the rock physics diagnostics rests on the data collected from the well sites. An important application of this model is to predict the petrophysical properties from both seismic and elastic attributes together. The porosity determination from seismic based acoustic impedance and V_P_/V_S_ ratio is an important objective of this study (Fig. 13, Eqs. (27), (28)). Therefore, two different equations developed for porosity estimation depending on measured acoustic impedance and V_P_/V_S_ ratio by using linear regression method Eqs. (27), (28) which helps to differentiate various facies, namely shale facies, water sand facies and gas sand facies in a cross plot of rock physics domain, as verified with water saturation color bar in (Fig. 11D). Furthermore, these two equations (Eqs. (27), (28)) can enhance the interpretation of seismic data by converting acoustic impedance and the V_P_/V_S_ ratio into porosity estimates. In Fig. 13, the cross plot suggest that the porosity strongly correlated with acoustic impedance. According to Mavko et al.^83^, the porosity slope becomes steeper when it changes with diagenesis and horizontal when porosity changes for grain size, which also confirmed by rock physics diagnostic in (Fig. 13).

**Table 4:** The values of elastic properties of fluids and minerals for rock physics modeling^77,83–86^.

Minerals/fluids	Density (g/cm^3^)	Bulk modulus (GPa)	Shear modulus (GPa)
Quartz	2.65	37	44
Clay	2.7	15	5
Water	1.1	2.8	–
Gas	0.2	0.6	–

27 $$\:\text{P}\text{o}\text{r}\text{o}\text{s}\text{i}\text{t}\text{y}=0.575-4.214\:\times\:{10}^{-5}\:\times\:\text{I}\text{P}$$

28 $$\:\text{P}\text{o}\text{r}\text{o}\text{s}\text{i}\text{t}\text{y}=0.51-0.25\times\:\:\frac{{V}_{P}}{{V}_{S}}$$

## Conclusion

This study provides a comprehensive characterization of the deep Mangahewa Formation reservoir sandstones (3200–4000 m depth) in the Pohokura gas field. By integrating petrophysical and rock physics analyses (RPA) with a focus on rock physics templates (RPTs) and models (RPMs), the research effectively delineates the reservoir’s elastic properties and lithologic facies. The Mangahewa sandstones exhibit elastic behavior consistent with the stiff sand model, showing compressional sonic velocities of 4100–5000 m/s and effective porosities of 8–19%. High correlations between measured and modeled compressional (V_P_) and shear (V_S_) velocities, as well as densities (97, 94, and 99%, respectively), underscore the reliability of the model. Additionally, the observed increase in acoustic impedance with decreasing porosity suggests diagenetic effects on reservoir properties. The application of RPTs, particularly in the V_P_/V_S_ versus acoustic impedance domain, effectively differentiates gas sands, water sands, and shale facies, reducing uncertainty in fluid and lithology interpretation. This detailed analysis enhances understanding of the subsurface heterogeneity in the Mangahewa Formation, supporting more accurate hydrocarbon prospect evaluation within the Pohokura gas field and other gas fields in the Taranaki Basin.

## Data Availability

The datasets generated during and/or analyzed during this study may be obtained on reasonable request from the corresponding author.
